# A Booster Vaccine Expressing a Latency-Associated Antigen Augments BCG Induced Immunity and Confers Enhanced Protection against Tuberculosis

**DOI:** 10.1371/journal.pone.0023360

**Published:** 2011-08-17

**Authors:** Bappaditya Dey, Ruchi Jain, Umesh D. Gupta, V. M. Katoch, V. D. Ramanathan, Anil K. Tyagi

**Affiliations:** 1 Department of Biochemistry, University of Delhi South Campus, New Delhi, India; 2 National JALMA Institute for Leprosy and Other Mycobacterial Diseases, Agra, Uttar Pradesh, India; 3 Department of Clinical Pathology, Tuberculosis Research Center, Chennai, Tamil Nadu, India; Indian Institute of Science, India

## Abstract

**Background:**

In spite of a consistent protection against tuberculosis (TB) in children, *Mycobacterium bovis* Bacille Calmette-Guerin (BCG) fails to provide adequate protection against the disease in adults as well as against reactivation of latent infections or exogenous reinfections. It has been speculated that failure to generate adequate memory T cell response, elicitation of inadequate immune response against latency-associated antigens and inability to impart long-term immunity against *M. tuberculosis* infections are some of the key factors responsible for the limited efficiency of BCG in controlling TB.

**Methods/Principal Findings:**

In this study, we evaluated the ability of a DNA vaccine expressing α-crystallin- a key latency antigen of *M. tuberculosis* to boost the BCG induced immunity. ‘BCG prime – DNA boost’ regimen (B/D) confers robust protection in guinea pigs along with a reduced pathology in comparison to BCG vaccination (1.37 log_10_ and 1.96 log_10_ fewer bacilli in lungs and spleen, respectively; *p*<0.01). In addition, B/D regimen also confers enhanced protection in mice. Further, we show that B/D immunization in mice results in a heightened frequency of PPD and antigen specific multi-functional CD4 T cells (3^+^) simultaneously producing interferon (IFN)γ, tumor necrosis factor (TNF)α and interleukin (IL)2.

**Conclusions/Significance:**

These results clearly indicate the superiority of α-crystallin based B/D regimen over BCG. Our study, also demonstrates that protection against TB is predictable by an increased frequency of 3^+^ Th1 cells with superior effector functions. We anticipate that this study would significantly contribute towards the development of superior booster vaccines for BCG vaccinated individuals. In addition, this regimen can also be expected to reduce the risk of developing active TB due to reactivation of latent infection.

## Introduction

The success of *Mycobacterium tuberculosis* as a human pathogen relies immensely on its ability to subvert the host immune responses and persist in a dormant state. *M. bovis* Bacille Calmette-Guerin (BCG) is currently the only tuberculosis (TB) vaccine approved for human use. It has been used to vaccinate more than 4 billion individuals worldwide and still continues to be a part of the childhood immunization program in most of the countries due to its ability to impart effective protection against TB in children [1]. However, the protective immunity generated by BCG wanes off with age and its efficacy against the disease has been less than satisfactory in adults and older individuals. Besides, the inability of BCG to provide sterilizing immunity at the time of primary infection leads to an enormous reservoir of asymptomatically infected individuals worldwide (∼2 billion). These latently infected individuals have a persistent risk of developing clinical disease due to endogenous reactivation if the immune system is compromised due to several reasons such as HIV infection, malnourishment etc. [2], [3]. Latency-associated antigens are expressed by *M. tuberculosis* while adapting to long-term persistence; however, BCG being an attenuated strain does not persist long enough to express these antigens. Thus, despite sharing a vast repertoire of antigens with *M. tuberculosis,* BCG fails to elicit an efficient response against these latency antigens [4], [5], [6]. Hence, the latency-associated antigens are attractive targets for developing booster vaccines to enhance the protective efficacy of BCG [6].

In the last five years, there has been a substantial progress in the development of new TB vaccines and many of these have already found their way into clinical trials or pre-clinical development [7]. However, most of these vaccines are based on the antigens that are highly immunodominant and are recognized by the immune system during the early stages of *M. tuberculosis* infection [8], [9], [10], [11]. There are very few vaccine studies employing antigens expressed preferentially during adaptation of bacteria to long-term persistence [6], [12], [13], [14].

Previously, we have reported the development of DNA vaccine (DNAacr) expressing α-crystallin (a*cr*, HspX, *Rv2031c*) [14] – a key component of the dormancy regulon and one of the most abundantly produced proteins during exposure of *M. tuberculosis* to hypoxia, nutrient starvation as well as in the late stages of infection [4], [5], [6]. We showed that this DNA vaccine induces Th1 response and protects guinea pigs against *M. tuberculosis* infection, even though, it could not surpass the protective efficacy of BCG, when employed alone in a prophylactic mode [14]. Recently, we also showed that DNAacr efficiently boosts the immunity following vaccination with a recombinant BCG vaccine over-expressing α-crystallin [15]. However, as majority of global population represents BCG vaccinated individuals, in this study, we have evaluated the ability of a booster DNA vaccine expressing α-crystallin to strengthen the immune responses and enhance and prolong the protective efficiency of BCG. We show that a booster vaccine targeting a latency antigen can substantially enhance the protection imparted by BCG vaccine. We also show a distinct association of multi-functional T cell responses with the vaccine-induced protection.

## Results

### Enhanced protection by BCG prime-DNAacr boost regimen

**Figure 1:**
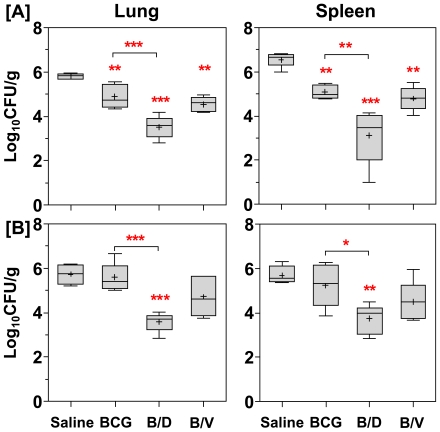
Enhanced protection against *M. tuberculosis* challenge by DNAacr boosting subsequent to BCG vaccination. For evaluation of protective efficacy two guinea pig experiments were performed. The figure depicts the bacillary load in the lungs and spleen of vaccinated and saline treated guinea pigs at (A) 10 weeks (Exp-I, n = 5) and (B) 16 weeks (Exp-II, n = 6) post-infection. Immunization with B/D regimen resulted in a significantly lower bacillary load in lungs and spleen, when compared to both BCG and saline groups. Log_10_ CFU were measured and graphically represented by box plot, wherein median values are denoted by horizontal line, the mean is represented by ‘+’, inter quartile range by boxes, and the maximum and minimum values by whiskers. The lower limit of detection was 1.0 log_10_ CFU/g of tissue and animals with undetectable bacilli were allotted a CFU value of 1.0 log_10_/g. B/D, BCG prime – DNAacr boost and B/V, BCG prime – vector boost. *, *p*<0.05, **, *p*<0.01, ***, *p*<0.001, when compared to the saline group or as indicated (One-way ANOVA).

To evaluate the protective efficacy of ‘BCG prime-DNAacr boost’ regimen (B/D), at 12 weeks after the primary immunization, guinea pigs were infected with *M. tuberculosis* by aerosol route and lung and spleen bacillary load were determined at 10 (Exp-I) and 16 weeks (Exp-II) post-infection. At 10 weeks post-infection (Fig. 1A), BCG vaccination resulted in a significantly reduced bacillary load in the lungs and spleen when compared to the unvaccinated animals (0. 94 log_10_ and 1.48 log_10_ fewer bacilli, respectively, *p*<0.05). However, when DNAacr was employed as a booster subsequent to BCG priming, the regimen conferred robust protection as was evident from a remarkable reduction in the bacillary load in the lungs and spleen (2.31 log_10_ and 3.44 log_10_ fewer bacilli, respectively, *p*<0.01), when compared to the unvaccinated animals (Fig. 1A). Moreover, the magnitude of protection by B/D regimen was especially noteworthy, as it resulted in 1.37 log_10_ and 1.96 log_10_ fewer bacilli in lungs and spleen, respectively in comparison to BCG vaccination (*p*<0.01), (Fig. 1A).

In Exp- II, when the post-challenge period was prolonged to 16 weeks, one third of the saline treated animals (2/6) succumbed to the disease. In the BCG vaccinated animals, although no mortality was observed, the bacillary load in the lungs and spleens of these animals was observed to be as much as in the case of unvaccinated animals (surviving ones) indicating a considerable decline in the protection conferred by BCG vaccination on extending the post-challenge period to 16 weeks (Fig. 1B). However, B/D regimen continued to provide a superior protection, as was evident from a significantly reduced bacillary load in the lungs and spleen when compared with the BCG vaccination (by 2.01 log_10_ and 1.47 log_10_, *p*<0.05, respectively; *p*<0.05). The influence of boosting was found to be antigen specific, since, boosting with vector DNA (B/V) did not show any significant improvement over BCG immunization (Fig. 1B).

### Reduced pathology following B/D vaccination

**Figure 2:**
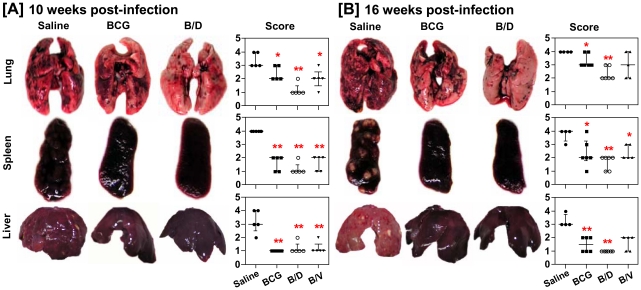
Influence of vaccination on gross pathology following *M. tuberculosis* infection. The figure depicts representative photographs and graphical depiction of gross scores of lung, liver and spleen of guinea pigs at (A) 10 weeks (n = 5) and (B) 16 weeks (n = 5) post-infection. Based on the extent of involvement, number and size of tubercles, areas of inflammation and necrosis, gross pathological scores were graded from 1 to 4. Each point represents score for an individual animal and the bar depicts median (± inter-quartile range) for each group, *p<0.05 and **p<0.01, when compared to the saline group (Mann-Whitney *U* test).

On examining the influence of vaccination on pathological damage in various organs of guinea pigs at 10 weeks post-infection, we observed that unvaccinated animals exhibited severe pathology in lungs, liver and spleen characterized by the presence of numerous large and small sized tubercles (Fig. 2A). Vaccination of animals with BCG and B/V resulted in significantly reduced gross lesions in lungs, liver and spleen, when compared to the unvaccinated animals. However, B/D vaccination imparted even a more pronounced effect on pathology, such that, the majority of animals vaccinated with this regimen exhibited only a minimal pulmonary involvement along with markedly reduced gross lesions in spleen when compared with BCG and B/V vaccinated animals (Fig. 2A).

**Figure 3:**
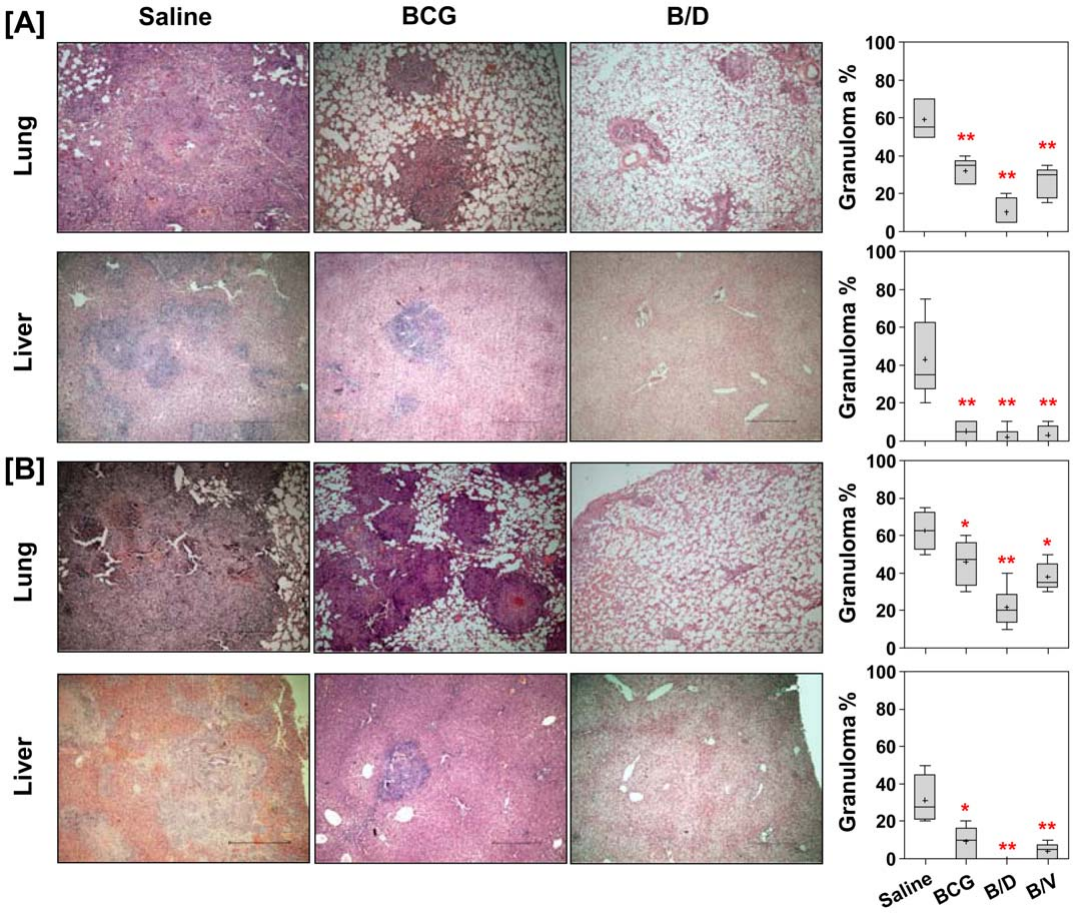
Vaccination with B/D regimen reduces granulomatous inflammation in *M. tuberculosis* infected guinea pigs. The figure depicts representative photomicrographs (4×) of H&E stained lung and liver sections of guinea pigs at (A) 10 weeks (n = 5) and (B) 16 weeks (n = 5) post-infection. H&E stained lung and liver sections were examined by light microscopy. Lungs: saline group exhibited multi-focal coalescing granulomas with extensive necrosis; BCG group showed discrete granulomas with or without central necrosis and B/D group exhibited negligible and diffused aggregates of inflammatory cells. Liver: saline group showed multiple granulomas, BCG vaccinated animals exhibited mild inflammation and B/D vaccinated animals exhibited no evident signs of inflammation. Scale bar represents 2 mm. Pulmonary and hepatic consolidation were graphically represented as % granuloma by box plot (notations are described in the legend of Fig. 1), *p<0.05 and **p<0.01, when compared to the saline group (Mann-Whitney *U* test).

Histopathological analysis of lung and liver sections further substantiated the gross pathological observations (Fig. 3A). Unvaccinated animals exhibited wide spread granulomatous inflammation with necrosis occupying 55% of the lung sections. Vaccination with BCG, B/D as well as B/V regimens resulted in a significantly reduced granulomatous inflammation in lungs (*p*<0.01). However, in contrast to BCG and B/V vaccinated animals which, showed the presence of granulomas covering 35% and 30% of the lung sections, respectively, animals vaccinated with B/D regimen exhibited well-preserved alveolar spaces with only a few scattered areas of diffused infiltration in peribronchial and perivascular areas (5%) (Fig. 3A). Similarly, in liver, the unvaccinated animals exhibited effacement of a large proportion of hepatic parenchyma by multi-focal necrotic granulomas covering almost 35% of the liver sections. BCG and B/V vaccinated animals exhibited minimal granulomatous inflammation in liver (5%). However, architecture of hepatic lobules was completely preserved in case of B/D vaccinated animals with no evident sign of inflammation (Fig. 3A).

Similarly, at 16 weeks post-infection, as expected, lungs and liver of unvaccinated animals exhibited extensive multi-focal coalescing granulomas with prominent central coagulative necrosis. In comparison, BCG as well as B/V vaccinated animals exhibited the presence of scattered areas of inflammation with discrete as well as coalescing granulomas with small necrotic centers in lungs (Fig. 2B and 3B). The liver of these animals, however, exhibited a significantly reduced inflammation. In contrast, B/D vaccinated animals exhibited only a negligible inflammation in lungs. In addition, the liver of these animals was also completely protected against pathological damage (Fig. 2B and 3B).

**Figure 4:**
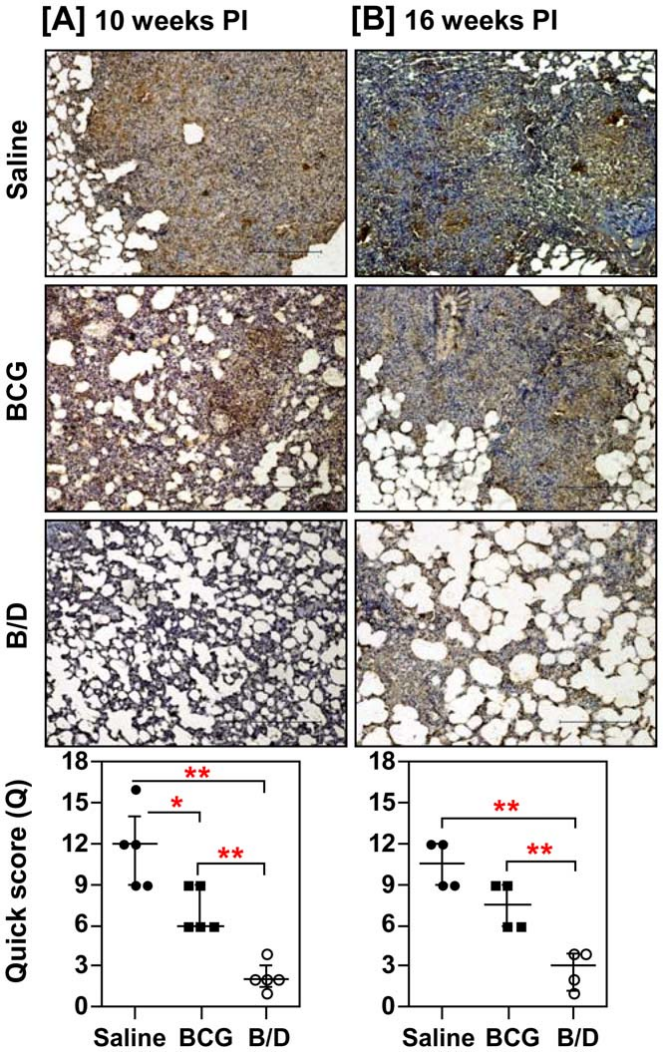
Influence of vaccination on the antigen load in pulmonary granulomas post-infection. The representative photomicrographs (10×) of 5 µm lung sections showing immuno-histochemical staining (brown color) for Ag85 complex proteins in pulmonary granulomas at (A) 10 weeks (Exp-I, n = 5) and (B) 16 weeks (Exp-II, n = 4) post-infection. Unvaccinated animals exhibited extensive staining surrounding and within the granulomatous areas; BCG vaccinated animals exhibited a moderate antigen staining and B/D vaccinated animals exhibited no evident sign of antigen staining in conjunction with negligible granulomatous inflammation. Scale bar represents 1 mm. Extent (Q) of staining was measured [Quick score (Q) = intensity (I) X area (A) of staining] and represented graphically as median (± inter quartile range). *, *p*<0.05 and **, *p*<0.01 (Mann-Whitney *U* test).

**Figure 5:**
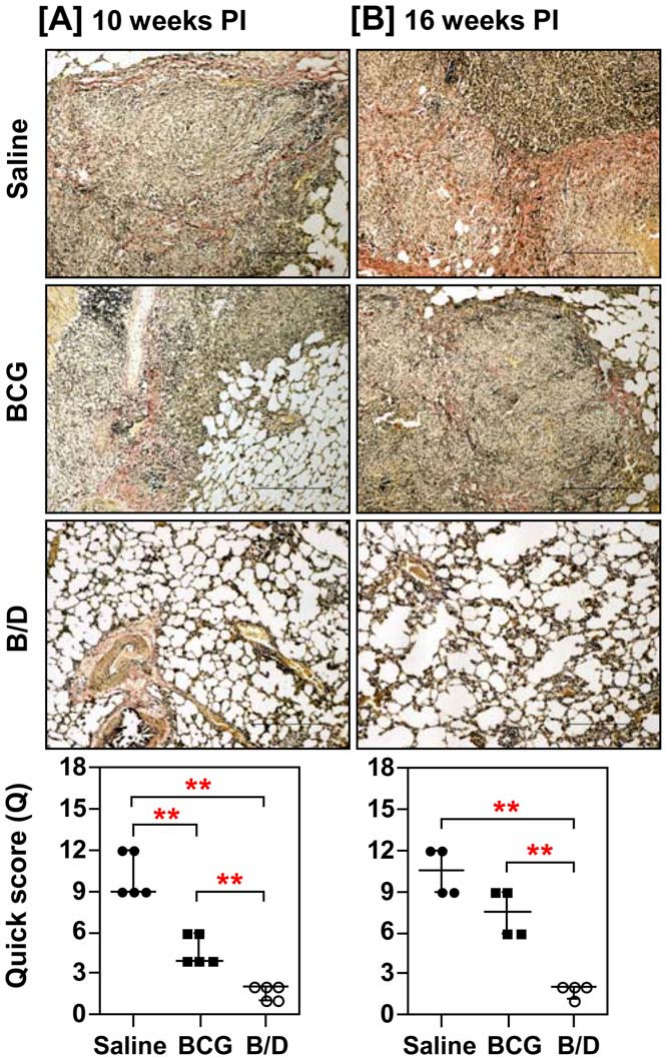
Influence of α-crystallin based prime boost vaccination on pulmonary fibrosis following *M. tuberculosis* infection. The figure depicts representative photomicrographs (10×) of Van Gieson stained lung sections of guinea pigs euthanized at (A) 10 weeks and (B) 16 weeks post-infection. Saline group exhibited extensive fibrosis or collagen deposition (red color) in granulomatous lesions; BCG group exhibited increased fibrosis with time and B/D group showed complete disease resolution with no evident sign of fibrosis. Scale bar represents 1 mm. Extent (Q) of pulmonary fibrosis was measured by light microscopy [Q = Intensity (I) X area (A) of staining] and represented graphically as median (± inter quartile range). B/D, BCG prime – DNAacr boost. **, *p*<0.01 (Mann-Whitney *U* test).

In parallel, we also determined the antigen load in lung tissues by *in situ* localization of Ag85 complex proteins, which serve as a marker for live bacilli, dead bacilli, bacillary remnants as well as secreted antigens [16]. We observed a strong positive correlation between antigen load and bacillary count (r = 0.78, *p*<0.001). Particularly, in concordance with the reduced bacillary load, animals immunized with B/D regimen exhibited minimal antigen load both at 10 and 16 weeks post-infection (Fig. 4). Commensurate with the extensive granulomatous inflammation, unvaccinated animals exhibited widespread fibrosis in and around the pulmonary granulomas causing loss of alveolar structure (Quick score, Q = 9 and 10.5, at 10 and 16 weeks, respectively) (Fig. 5). BCG vaccinated animals exhibited a moderate collagen staining at both the time points (Q = 4 and 7.5, at 10 and 16 weeks, respectively). In contrast, animals vaccinated with B/D regimen exhibited only a weak collagen staining in the lungs (Q = 2, at both the time points), when compared with the BCG vaccinated animals (*p*<0.05), (Fig. 5). The extent of collagen deposition also showed a strong positive correlation with the bacillary load (r = 0.879, *p*<0.001) and granulomatous inflammation (r = 0.876, *p*<0.001).

Altogether, these observations suggest that vaccination with B/D regimen, in addition to exerting an efficient control on bacillary multiplication and imparting a markedly superior protection against pathological damage and fibrosis, the regimen was also efficient in meditating antigenic clearance from the site of infection.

### Induction of multifunctional CD4 Th1 cell response by B/D regimen confers enhanced protection in murine model

The non-availability of immunological reagents, a persistent caveat of guinea pig model, prohibited us from evaluating the T cell effector functions and their association with the protection imparted by various vaccination regimens. Hence, we evaluated the protective efficacy of B/D regimen in murine model also along with the associated immune responses.

**Figure 6:**
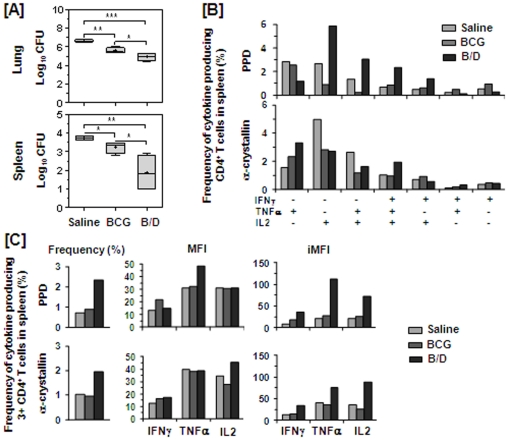
B/D vaccination confers enhanced protection against *M. tuberculosis* challenge in mice and induces heightened multifunctional CD4 T cell response. The figure depicts (A) the bacillary load in lungs and spleen of mice (n = 4) at 4 weeks post-infection and (B–C) multifunctional T cell response at 12 weeks post-immunization. Mice experiment was performed once. (A) Log_10_ CFU is represented by box plot (notations are described in the legend of Fig. 1). The lower limit of detection was 1.0 log_10_ CFU/g of tissue and animals with undetectable bacilli were allotted a CFU value of 1.0 log_10_/g. *, *p*<0.05, **, *p*<0.01 and ***, *p*<0.001. (One-way ANOVA). (B–C) T lymphocytes were purified from PPD and α-crystallin stimulated splenocytes (pooled from four mice per group) and stained for CD4 T cell surface marker along with intracellular cytokine staining for IFNγ, TNFα and IL2 followed by FACS analysis. (B) Represents frequency (%) of PPD and α-crystallin specific CD4 T cells expressing each of the seven combinations of IFNγ, TNFα and IL2. (C) Represents frequency of PPD and α-crystallin specific 3^+^ CD4 T cells along with MFI and iMFI for IFNγ, TNFα and IL2. B/D: BCG prime – DNAacr boost regimen.

At 4 weeks post *M. tuberculosis* infection, we observed a significantly reduced bacillary load in the lungs and spleen of BCG vaccinated animals when compared with the unvaccinated animals (1.02 log_10_ and 0.48 log_10_ fewer bacilli in the lungs and spleen, respectively), (Fig. 6A). A strikingly higher reduction in the bacillary load was observed in case of animals vaccinated with B/D regimen in comparison to the unvaccinated animals (by 1.69 log_10_, *p*<0.001 and 1.84 log_10_, *p*<0.01 fewer bacilli, respectively). Even in comparison to the BCG vaccinated animals, the animals vaccinated with B/D regimen exhibited a significantly reduced bacillary load (by 0.67 log_10_ and 1.35 log_10_ fewer bacilli, respectively, *p*<0.05), (Fig. 6A) indicating thereby that this regimen provides a superior protection in comparison to BCG.

Further, we analysed the quality of T cell responses by measuring the cytokine production on a per-cell basis by FACS following a six-hour stimulation of splenocytes with PPD or α-crystallin. T cells were purified, stained for CD4 T cell surface marker along with IFNγ, TNFα, and IL2 and were subjected to FACS analysis. Quality of immune responses was defined on the basis of expression of various combinations of these cytokines as described by Seder *et al* [17]

At 12 weeks post-primary immunization (at the time of infection), although quantitatively, both BCG and B/D vaccinated animals exhibited a comparable frequency of CD4 T cells (data not shown), qualitatively, a marked functional heterogeneity was observed in terms of their ability to produce IFNγ, TNFα and IL2 (Fig. 6B). Notably, B/D vaccinated animals exhibited a markedly higher frequency of PPD specific 3^+^ (IFNγ^+^TNFα^+^IL2^+^) and 2^+^ (TNFα^+^IL2^+^ and IFNγ^+^IL2^+^) CD4 T cells when compared with the BCG vaccinated animals (Fig. 6B). In addition, 3^+^ CD4 T cells specific to PPD in B/D vaccinated animals also exhibited a markedly higher iMFI for all the three cytokines, when compared with the BCG vaccinated animals (Fig. 6C). In contrast, BCG vaccinated animals exhibited a higher 1^+^ CD4 T cell response, with a characteristic preponderance of IFNγ^+^ and TNFα^+^ cells (Fig. 6B). Moreover, B/D vaccination also boosted the multifunctional 3^+^ CD4 T cell response specific to α-crystallin antigen (Fig. 6C). These observations indicate that the enhanced protection by B/D regimen is associated with augmentation of 3^+^ multifunctional CD4 T cell response.

## Discussion

BCG has been the most widely used vaccine over the last several decades with more than 4 billion Individuals vaccinated with it worldwide. However, clinical trials and subsequent meta-analysis revealed its general ineffectiveness against TB in adults [1]. As a result, several laboratories all over the world have now directed their focus towards the development of new candidate vaccines or vaccination regimens that can improve upon the protection conferred by BCG vaccine [7]. According to one of the proposed mechanisms, as the individual reaches 10 to15 years of age, the memory immunity generated by BCG declines resulting in a gradual loss of efficacy of BCG [18]. Thus, introduction of a booster vaccine based on a candidate antigen specifically recognized by BCG induced immunity emerged as one of the most successful approaches [18]. Among the various boosting agents, vaccines of genetic nature such as DNA vaccines have emerged as one of the most promising advancements in the field of vaccine discovery. Immunization with DNA is known to direct the immune system towards stronger and persistent cellular and humoral immune responses [19]. This is especially advantageous in the context of TB as the protective immunity against *M. tuberculosis* is primarily dependent on the cell-mediated immune responses [20]. Several of the DNA vaccine candidates, when used in homologous prime boost regimen have exhibited a considerable potential in mouse model of tuberculosis, such as, those expressing Ag85A (mycolyl transferase) [21], ESAT-6 [14], PstS-1, PstS-3 [22] and hsp65 [23], however, the protection conferred by these vaccines was generally lower and rarely equal to BCG [24], [25]. In addition, they were found to be ineffective, when evaluated as a standalone vaccine in a more stringent guinea pig model [21], [26]. To date, a very few DNA vaccines have proved successful when employed in a heterologous prime boost regimen with BCG, such as DNA vaccines based on a fusion protein 72f and Hsp65 antigen based DNA vaccine [27], [28].

In this study, we show that α-crystallin (hspx, *Rv2031c*) based DNA vaccine booster subsequent to BCG priming is highly effective in improving the protection conferred by BCG. This heterologous prime boost regimen provides a superior and extended protection against *M. tuberculosis* infection in guinea pigs as well as in mice. According to our previous findings, α-crystallin based DNA vaccine, although, failed to surpass the protection conferred by BCG vaccine, when used as a standalone vaccine [14], it is was able to substantially improve the protection conferred by a recombinant BCG vaccine over-expressing this antigen [15]. Our current findings are thus, all the more important as they signify that the meager immune responses generated by BCG specific to α-crystallin can be substantially boosted by this DNA vaccine resulting in enhanced and extended protection superior to BCG.

Our observations in murine model further highlight the association between the enhanced protection by B/D regimen and the production of a heightened multi-functional (3^+^) CD4 T cell responses along with production of overall larger quantities of IFNγ, TNFα and IL2 by these T cells. Furthermore, an increased frequency of 2^+^ CD4 T cells simultaneously producing TNFα and IL2 in case of B/D regimen indicates the generation of a greater central memory response in comparison to BCG. Though, more extensive phenotyping of these 2^+^ T cells would have helped, previous studies have proposed that while, the central memory T cells produce IL2 alone or IL2 along with TNFα, the effector memory T cells produce IFNγ either alone or in combination with IL2 and TNFα [17]. A lower degree of protection by BCG vaccination despite the production of a comparable frequency of CD4 T cells further illustrates that it is the functionality of T cells and their ability to produce a broader repertoire of cytokines, which plays a major role in the degree of protection conferred by a vaccine. These observations are also in agreement with previous studies, which have shown that T cells with multiple effector functions, such as concomitant production of IFNγ, TNFα and IL2 are functionally superior to their counterparts producing only a single cytokine [29]. In addition, induction of these multifunctional T cells by vaccine regimens have also been shown to be a potential correlate of protection against tuberculosis as well as against other bacterial and viral diseases [30], [31], [32]. Although, the CD4 Th1 cell responses in this study were analysed on pooled samples and thus, need to be interpreted cautiously, enhanced protection observed in case of B/D vaccination does indicate a crucial role of these multifunctional CD4 Th1 cells in conferring long-term protection against *M. tuberculosis* infection. Moreover, caution should be exercised to extrapolate the mouse immunogenicity data to guinea pigs due to inherently different responses of the two species to *M. tuberculosis* infection until the immunological reagents for guinea pigs become available and the involvement of multifunctional T cells in mediating protection is formally demonstrated in this species.

‘BCG prime-DNAacr boost’ regimen should be advantageous in terms of several clinical applications. Firstly, in view of the large population vaccinated with BCG, DNAacr boosting can be expected to significantly enhance and prolong the protection conferred by BCG vaccine. This prospect gains further strength from an important finding, wherein Pathan *et al*. reported that the effect of an efficient booster vaccine remains unaltered irrespective of the time span between the primary BCG vaccination and boosting; a comparable enhancement in immune responses was observed, whether the booster was administered a few months or many years after BCG vaccination [33]. Secondly, since, the valuable attributes of BCG vaccination are preserved in this strategy, DNAacr, as a booster vaccine would simplify the issues related to the clinical testing of this regimen without hampering the childhood immunization program. Thirdly, induction of an efficient immune response by this regimen against α-crystallin can also be speculated to reduce the occurrence of latent TB infection by promoting clearance of the dormant bacilli. While, further studies would be required to demonstrate these plausible advantages, the importance of α-crystallin in protection against TB is further substantiated by the observation in our laboratory, wherein α-crystallin based DNA vaccine, when evaluated in therapeutic mode as an adjunct to chemotherapy in a modified Cornell model, was found to effectively promote bacillary clearance, reduce the time of chemotherapy as well as confer a significant protection against reactivation (unpublished observations). Moreover, a recent study by Aagaard *et al* has also demonstrated that a multi-stage vaccine, which is based on combination of early and latency-associated antigens not only serves as an efficient booster to BCG vaccine conferring superior protection in a prophylactic mode but also provides a significant degree of protection against reactivation when employed alone as an adjunct to chemotherapy [6]. Altogether, the success of α-crystallin based DNA vaccine in this study would not only contribute towards the development of efficient booster vaccines for BCG but would also encourage the designing and evaluation of TB vaccines for prophylactic as well as therapeutic purposes based on latency antigens.

## Materials and Methods

### Ethics statement

Guinea pig experiments were reviewed and approved by the Institutional Animal Ethics Committee of National JALMA Institute for Leprosy & Other Mycobacterial Diseases, Agra, India (Permit number: 101/1997/CPCSEA). Mice experiments were reviewed and approved by the Institutional Animal Ethics Committee of University of Delhi South Campus, New Delhi, India (Permit number: 159/1999/CPCSEA). All animals were routinely cared for according to the guidelines of CPCSEA (Committee for the Purpose of Control and Supervision on Experiments on Animals), India.

### Bacterial strains, preparation of vaccines for immunization and animals

*M. bovis* BCG (Danish strain, BCG laboratories, Chennai, India) and *M. tuberculosis* (H37Rv strain, ATCC no. 25618, AIIMS, New Delhi, India) were grown to mid-log phase in Middle Brook 7H9 media and stocks were prepared as described [16]. DNA vaccine, pAK4-αcry - expressing α-crystallin was prepared as described [14]. Outbred guinea pigs (Dunkin Hartley strain, female, 200–300 g) were procured from Disease Free Small Animal House Facility, HAU, India and maintained in the BSLIII facility at National JALMA Institute for Leprosy & Other Mycobacterial Diseases, Agra, India. Mice (Balb/c strain, female, 6–8 weeks) were procured from National Centre for Laboratory Animal Science, National Institute of Nutrition, Hyderabad, India and were maintained in the BSLIII facility at University of Delhi South Campus, New Delhi, India.

### Immunization of guinea pigs and evaluation of protective efficacy against *M. tuberculosis* infection

For evaluation of protective efficacy, two guinea pig experiments were performed. In each experiment, guinea pigs were immunized with (i) 5×10^5^ CFU of BCG (in 100 µl of saline) by i.d. route, (ii) BCG once, followed by a booster dose of DNAacr or vector (100 µg in 100 µl of saline) by i.m. route at 6 weeks (B/D or B/V) and (iii) 100 µl of saline by i.d. route (control group). Guinea pigs were challenged 12 weeks after the primary immunization with 50–100 bacilli of *M. tuberculosis* H37Rv *via* the respiratory route in an aerosol chamber (Glasscol Inc.). Animals were euthanized at 10 weeks post-infection in Exp-I and at 16 weeks post-infection in Exp-II by intraperitoneal injection of Thiopentone sodium (100 mg/kg body weight) (Neon Laboratories Ltd.). After dissecting the animals, lungs, liver and spleen were scored for pathological changes as described previously [16], [34]. For histopathological evaluation, all three lung lobes (right caudal, middle and cranial) and a portion of left dorsal lobe of liver were removed and fixed in 10% buffered formalin. Left caudal lung lobe and cranial portion of spleen were aseptically removed for the measurement of bacillary load. Lung and spleen bacillary load was measured along with the evaluation of histopathological changes as described [16]. The detection limit in case of both lung and spleen CFU was 1.0 log_10_ CFU/g.

### Evaluation of protective efficacy and immune responses in mice

Protection studies in mice were carried out once similarly as described for guinea pigs. To recapitulate, briefly, mice in groups of 4 were immunized and infected following the same protocol as used for guinea pigs. Immune responses were measured at 12 weeks post-immunization (at the time of infection). For evaluation of protective efficacy, bacillary load in lungs and spleen was determined at 4 weeks post-infection.

### Cell Isolation, stimulation and flow cytometry for the assessment of CD4 T cell responses

At 12 weeks post-primary immunization, T cell responses were assessed by intracellular cytokine staining of CD4 T cells for IFNγ, TNFα, and IL2. Briefly, spleen cells were isolated as described [35] and cultured in RPMI-GlutaMAX™ (containing 10% heat inactivated FBS and 1× antibiotic-antimycotic) (Invitrogen) in the presence of PPD (20 µg/ml) (Statens Serum Institut) or purified α-crystallin (40 µg/ml) for 2 hr at 37°C. Thereafter, GolgiStop was added and cells were incubated for an additional 4 hr before intracellular cytokine staining.

Following stimulation, cells were purified by using BD IMag™ Mouse T Lymphocyte Enrichment Kit. Briefly, a cocktail of biotinylated antibodies was added to the cells that simultaneously bind erythrocytes and leukocytes but not T lymphocytes. The cells were then washed to remove excess antibodies followed by the addition of BD IMag™ Streptavidin. Unlabelled T lymphocytes were then enriched by negative selection by placing them within the magnetic field of the BD IMagnet™. Cells were washed and incubated with BD Fc Block™ (CD16/CD32 mAb to block Fc binding). Subsequently, the cells were stained for cell surface marker, CD4 (FITC) along with intracellular cytokines, IFNγ (RPE), TNFα (PerCPCy5.5) and IL2 (APC) by using the BD Cytofix/Cytoperm kit. Cells were washed twice with Cytoperm and finally resuspended in sheath fluid. Cells (50,000–100,000) were acquired by using a FACS Calibur flow-cytometer (by using Cell Quest Pro software) and analyzed by using FlowJo software (Tree Star). Cell frequency, median fluorescence intensity (MFI) and integrated MFI (iMFI = % frequency X MFI) for different cytokines were calculated. For cell stimulation and FACS analysis, cells pooled from all 4 mice from a group were used. All the reagents for cell isolation and antibodies employed in FACS analysis were obtained from BD Pharmingen.

### Immunohistochemistry and image analysis

For *in situ* localization of *M. tuberculosis* antigens in the sections of guinea pig lungs, immunohistochemical (IHC) staining was carried out and analysed for area and intensity of staining as described earlier [16].

### Statistical analysis

For comparison of the CFU one-way ANOVA was carried out followed by Tukey test. Non-parametric Kruskal-Wallis test, followed by Mann-Whitney *U* test were employed for comparison of the quick score (Q) for IHC, gross pathological score and granuloma % across different groups. Correlation between different parameters was analysed by Spearman Rank Correlation test. Differences were considered significant, when *p*<0.05. For statistical analysis SPSS (Version. 10.0, SPSS Inc.) and Prism 5 (Version 5.01; GraphPad Software Inc.) were employed.
